# Uncommon late parietal wall complication of retained gallstones after laparoscopic cholecystectomy: a case report

**DOI:** 10.1093/jscr/rjag079

**Published:** 2026-02-19

**Authors:** Taher Laabidi, Aziz Atallah, Zied Hadrich, Mohamed Guelbi, Rached Bayar, Sahir Omrani

**Affiliations:** Department of Surgery, Mongi Slim Hospital, Marsa 2046, Tunisia; Department of Surgery, Mongi Slim Hospital, Marsa 2046, Tunisia; Department of Surgery, Mongi Slim Hospital, Marsa 2046, Tunisia; Department of Surgery, Mongi Slim Hospital, Marsa 2046, Tunisia; Department of Surgery, Mongi Slim Hospital, Marsa 2046, Tunisia; Department of Surgery, Mongi Slim Hospital, Marsa 2046, Tunisia

**Keywords:** laparoscopic cholecystectomy, retained gallstones, parietal abscess, late complication, case report

## Abstract

Retained gallstones after laparoscopic cholecystectomy (LC) are uncommon but can cause late abscesses or fistulas, sometimes years after surgery. Their recognition remains essential to avoid misdiagnosis. A 70-year-old man presented four years after LC with a right hypochondrial swelling. Imaging revealed a parietal abscess containing multiple gallstones. Percutaneous drainage followed by surgical extraction was performed. *Morganella morganii* was cultured, and the patient recovered uneventfully. Gallstone spillage during LC is frequent but rarely leads to clinical complications. Retained stones may act as foreign bodies and become infected, leading to chronic abscess formation. Imaging, particularly computed tomography, is crucial for diagnosis, and complete surgical removal is the cornerstone of treatment. Retained gallstones should be suspected in patients with unexplained parietal or intra-abdominal abscesses years after surgery. Prevention through meticulous technique and documentation is critical.

## Introduction

Laparoscopic cholecystectomy (LC) has become the gold standard for the management of symptomatic gallstones due to its safety, low morbidity, and short recovery time. Nevertheless, it is not devoid of complications. Gallbladder perforation with bile or stone spillage occurs in up to 40% of procedures, while 1%–2% result in unretrieved gallstones within the peritoneal cavity [1]. Although most of these stones remain clinically silent, 0.08%–0.3% can later cause significant complications such as abscesses, adhesions, or fistulas [2, 3].

The mechanism involves intraoperative gallbladder perforation with subsequent migration of stones along tissue planes, sometimes facilitated by infected bile or adhesions [4]. When infection is present, the stones can act as a persistent foreign body and serve as a nidus for chronic suppuration [5].

This case report highlights a rare presentation of a parietal wall abscess secondary to retained gallstones, appearing four years after an uneventful LC. We aim to underline the diagnostic difficulties and discuss management strategies in light of the current literature.

## Case presentation

A 70-year-old man presented with a painless swelling in the right hypochondrium, progressively enlarging over three months. He had undergone LC in 2019 for symptomatic cholelithiasis. The operative note was unavailable, but the early postoperative course had been uneventful.

**Figure 1:**
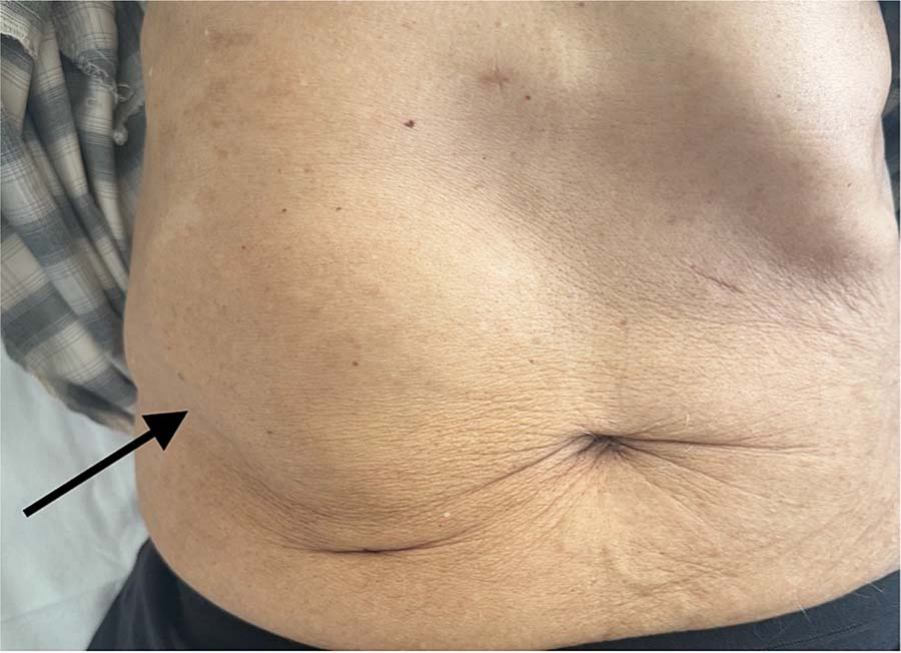
Clinical photograph of the abdomen demonstrating a localized swelling of the right hypochondrium (arrow), consistent with a parietal abdominal wall abscess.

Physical examination revealed a soft, fluctuant mass measuring approximately 8 cm in the right upper quadrant, without skin erythema or tenderness (Fig. 1). There were no systemic symptoms such as fever or weight loss. Laboratory investigations showed elevated inflammatory markers (white blood cells 20 000/mm^3^, CRP 210 mg/L), while liver and renal function tests were normal.

**Figure 2:**
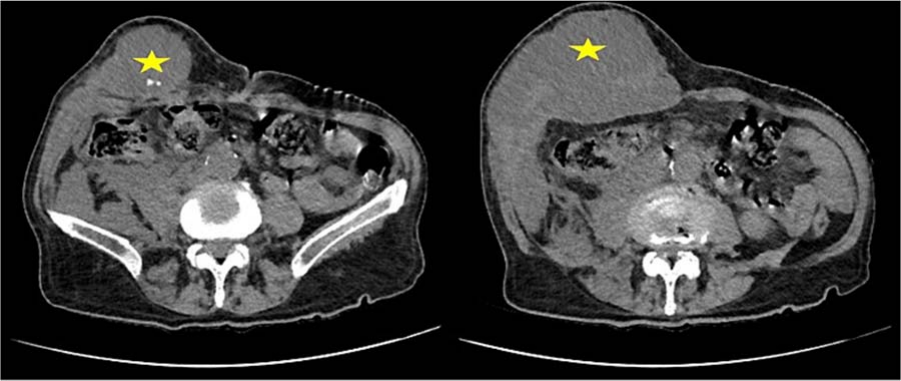
Contrast-enhanced CT scan of the abdominal wall showing a parietal abscess containing hyperdense foci consistent with retained gallstones (star).

Abdominal ultrasound revealed a heterogeneous parietal collection with internal hyperechoic foci. A contrast-enhanced computed tomography (CT) scan confirmed a large abscess (9 × 12 cm) within the abdominal wall, extending posteriorly, containing multiple calcified foci consistent with gallstones (Fig. 2). No biliary dilation or intra-abdominal collection was detected.

**Figure 3:**
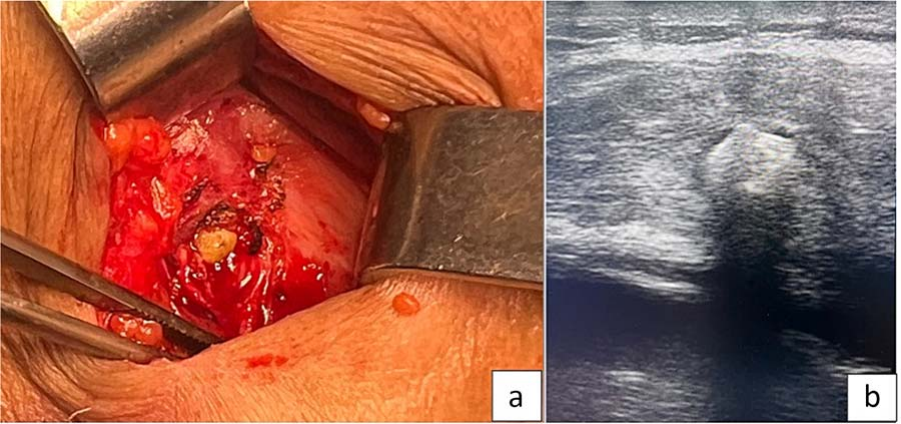
(A) Intraoperative image showing a parietal gallstone within the abdominal wall abscess cavity. (B) Intraoperative ultrasonography demonstrating a retained parietal gallstone.

A staged approach was chosen. Initial percutaneous drainage under imaging guidance was performed to control sepsis and decompress the collection. The pus was thick and purulent. After stabilization and normalization of inflammatory markers, surgical exploration via a right subcostal incision allowed evacuation of the abscess and removal of several gallstones (Fig. 3). The cavity was irrigated and drained. Intraoperative ultrasonography was used to accurately identify and ensure complete removal of all retained gallstones.

Microbiological culture grew *Morganella morganii*, sensitive to third-generation cephalosporins. The patient received 10 days of intravenous antibiotics followed by oral therapy.

Postoperative recovery was uneventful. The patient was discharged on postoperative Day 3. At 6-month follow-up, he remained asymptomatic, and control ultrasound confirmed complete resolution without recurrence.

## Discussion

Gallstone spillage during LC is frequent, occurring in 6%–40% of cases, yet only a small fraction lead to complications [1, 3]. The spilled stones may remain inert or, if infected, induce a chronic inflammatory response. Pigment stones are particularly prone to complications due to their high bacterial load and irritative properties [6].

The pathogenesis involves stone migration along peritoneal or fascial planes. Over time, this can lead to parietal or even thoracic localization. The delay in presentation is variable, ranging from a few months to several decades [7, 8]. In our case, the four-year interval highlights the insidious and delayed nature of this complication, contributing to diagnostic difficulty.

Clinical manifestations depend on the location of the retained stones. The most frequent presentations are intra-abdominal abscesses (subhepatic, subphrenic, retroperitoneal) and port-site infections [9]. Parietal wall abscesses remain exceptional, often mimicking tumors or hernias and leading to diagnostic confusion. Symptoms may be minimal, with only a localized mass and mild inflammatory response, as observed in our patient, which complicates diagnosis [10].

Radiological imaging plays a pivotal role. Ultrasound can suggest the diagnosis by showing hyperechoic foci with posterior shadowing, but CT is the gold standard [11]. CT not only confirms the presence of calcified stones but also delineates their relationship to adjacent structures, which was essential in our case to confirm the parietal origin and exclude intra-abdominal involvement, guiding surgical planning.

The bacterial profile mirrors that of infected bile. *Escherichia coli*, Klebsiella spp., Enterococcus spp., and *M. morganii* are the most common isolates [12]. These bacteria may remain dormant on the stone surface for years, protected by biofilm, and reactivate under favorable conditions [13]. In our patient, the isolation of *M. morganii* supports the hypothesis of secondary infection of retained stones from the initial cholecystectomy.

The treatment of retained gallstone complications should aim at both infection control and complete stone removal. Percutaneous drainage alone, while effective for temporary relief, is insufficient because the stones act as a persistent nidus for infection [14]. This may lead to recurrence or chronic suppuration if definitive treatment is delayed. Definitive management requires surgical evacuation and extraction of all stones, with debridement of the cavity.

The surgical approach depends on the location and extent of the abscess. Open surgery is preferred for large parietal or multiloculated abscesses, as in our case, while laparoscopic retrieval can be used for intra-abdominal collections [15]. Culture-guided antibiotic therapy should accompany the procedure for at least 10–14 days.

Prevention is essential. During LC, careful dissection and gallbladder handling can minimize perforation. In the event of stone spillage, thorough irrigation, suction, and retrieval of visible stones are mandatory. Importantly, documentation of intraoperative stone loss in the operative report and communication with the patient are crucial medico-legal safeguards. Such measures also facilitate early recognition of delayed complications.

A review of the literature identifies few reported cases of parietal wall abscesses due to retained stones. Zehetner *et al*. [1] described a series of 102 cases, with only seven involving the abdominal wall. Yao *et al*. [10] reported a similar case two years after LC. These data emphasize the rarity of isolated parietal involvement. Across reports, the delay from surgery to symptoms varied widely, from 6 months to 20 years, and *E. coli* or Klebsiella were the most frequent pathogens.

Our case aligns with these findings, but is distinguished by the isolated parietal localization and prolonged asymptomatic interval, confirming that even a single lost stone can cause delayed suppuration long after an apparently uncomplicated cholecystectomy. The rarity and nonspecific presentation often lead to misdiagnosis and inappropriate management, such as repeated drainage without definitive stone removal.

Prognosis after complete extraction is excellent. Recurrence or chronic sinus formation occurs only if stones remain or if the cavity is inadequately debrided [6]. Postoperative imaging is useful to ensure complete evacuation, especially when multiple stones are found intraoperatively. Close follow-up is recommended in cases with delayed presentation or atypical localization.

## Conclusion

Retained gallstones after LC are a rare but important cause of delayed abdominal or parietal abscesses. Computed tomography is key for diagnosis, and definitive management requires complete stone removal in addition to infection control. Careful surgical technique, thorough lavage, and proper documentation remain essential preventive measures.

## Data Availability

This published article includes all the required data.
